# A single intraarticular injection of a tranexamic acid-modified hyaluronic acid (HA/TXA) alleviates pain and reduces OA development in a murine model of monosodium iodoacetate-induced osteoarthritis

**DOI:** 10.3389/fphar.2024.1456495

**Published:** 2024-09-11

**Authors:** Sybille Brochard, Karim Boumédiene, Jéromine Mercier, Véronique Agin, Thierry Conrozier, Catherine Baugé

**Affiliations:** ^1^ UR7451 Bioconnect, Université de Caen Normandie, Caen, France; ^2^ Laboratoire de Rhumatologie Appliquée, Lyon, France; ^3^ INSERM U1237, Physiopathology and Imaging of Neurological Disorders, Université de Caen Normandie, Caen, France; ^4^ Department of Rheumatology, Hôpital Nord Franche-Comté, Belfort, France

**Keywords:** hyaluronic acid, tranexamic acid, controlled trial, rodents, osteoarthritis

## Abstract

**Rationale:**

Tranexamic acid (TXA) is a strong and specific plasminogen activator inhibitor with inhibitory effects on the matrix metalloproteases involved in the pathophysiology of osteoarthritis (OA) through targeting of the fibrinolysis pathway. In this study, we evaluated the analgesic and chondroprotective effects of a HA-tranexamic acid (HA/TXA) conjugate, compared to HA alone and placebo, in an animal model of knee OA.

**Methods:**

Knee OA was induced in 15 C^5^7 b l/6J mice by IA injection of 0.75 mg of Monosodium IodoAcetate (MIA). At day 28, the mice received 1 IA injection of 10 µL of saline (control-group), or of HA or of HA/TXA. Tactile sensitivity was assessed using von Frey filaments. Stimulations started at 1 g and increased until a response was obtained (up to 4 g). A response to the stimulus was counted if the animal withdrew its paw. If the animal responded to the 1 g stimulation, stimulation was reduced until the lack of response was observed (up to 0.2 g). At day 56, mice were euthanized for knee histological assessment. Cartilage degradation was assessed using the OARSI score. Statistical analysis was performed on GraphPad Prism 8.0.2 software. Kruskal–Wallis or Mann-Whitney tests were performed as appropriate.

**Results:**

Just before treatment administration, no intergroup difference in paw withdrawal threshold was observed. Throughout the experiment animals given saline and HA had a lower paw withdrawal threshold than those treated with HA/TXA (*p* < 0.01). In the control group OARSI score was 5.5 ± 0.6. In HA and HA + TXA treated mice the OARSI score was 3.2 ± 0.8 and 3.1 ± 0.5 (*p* < 0.01) showing that both treatments were able to reduce OA progression.

**Conclusion:**

In this animal model of MIA induced KOA, a single IA injection of a HA/TXA conjugate resulted in a greater efficacy on pain than both saline and HA. HA and HA/TXA exhibited chondroprotective effects compared to placebo.

## Introduction

Osteoarthritis (OA) is a leading cause of disability-adjusted life years (DALYs) (Cross et al., 2014). By 2050, it is estimated that there will be 642 million (95% CI 574–722) people suffering from knee OA (Steinmetz et al., 2023) resulting in an increased economic burden due to the growing number of joint arthroplasties (Jonsson et al., 2016). The etiology of OA is complex, involving advanced age and mechanical (i.e., excess weight, trauma), hereditary and environmental factors (Nicholls et al., 2017). Inflammation of the synovium plays a central role in the development and progression of OA. Multiple inflammatory mediators are involved, including interleukin (IL)-1β, IL-6, IL-17 and tumor necrosis factor α (TNF-α), the effects of which are mediated through the linkage with receptors, including CD44, HA-mediated motility receptor (RHAMM), and toll-like receptors (TLRs) (Dunn et al., 2009). IL-1β binds to receptors on articular cartilage chondrocytes and synovial cells and increases matrix metalloproteinases (MMPs) synthesis. Collagenases MMP-1 and MMP-13, by degrading type 2 collagen, play a major role in the hyaline cartilage extracellular matrix (ECM) breakdown. IL-1β also stimulates chondrocyte production of A Disintegrin And Metalloproteinase with Thrombospondin motifs (ADAMTS), a family of enzymes which degrade ECM aggrecans (Durigova et al., 2011; Massoud and Jian Q., 2008). The increased expression of MMPs in OA chondrocytes, and the better understanding of the pathways involved in their upregulation, has made MMPs interesting targets for OA treatment (Radwan et al., 2013). Non-proteolytic mechanisms of cartilage degradation can also contribute to the loss of ECM integrity. IL-1β also increases nitric oxide (NO) synthesis and decreases expression of superoxide dismutase and glutathione peroxidase, leading to an acceleration of the damaging effects of oxygen radicals on ECM (Kapoor et al., 2011). Lastly, it has recently been demonstrated that activation of fibrinolysis may promote OA development through multiple mechanisms, including the degradation of lubricin and cartilage proteoglycans and the induction of inflammatory and degradative mediators, suggesting that therapeutic targeting of the fibrinolysis pathway can prevent or slow development of OA. Indeed, in mice, genetic deficiency in the plasminogen gene *Plg* and pharmacological blockade of plasmin attenuate OA while genetic deficiency of plasminogen activator inhibitor (PAI) accelerates OA (Wang et al., 2024).

Currently, there is no effective treatment for this severe disease. The primary approach involves pain management and efforts to slow the disease’s progression (Sukhikh et al., 2021). Clinical treatment typically includes oral medications like non-steroidal anti-inflammatory (NSAID) drugs or using bioactive compounds from natural sources which have shown their potential due to their anti-inflammatory and antioxidant properties (Lee et al., 2021; Lee et al., 2022). However, long-term use of these drugs can cause damage to various organs, particularly the kidneys, gastrointestinal tract, and cardiovascular system (Cabassi et al., 2020) and there is no rigorous clinical proof of their actual effectiveness of natural compounds (Liu et al., 2018). Therefore, there is an urgent need to develop a treatment strategy that can alleviate osteoarthritis symptoms over an extended period with minimal side effects in clinical practice.

Hyaluronic acid (HA) intra-articular (IA) injections, also named viscosupplementation, are a treatment used worldwide for knee OA for more than 30 years (Conrozier et al., 2023). The rational of the use of IA-HA for treating OA is based on the evidence that HA, beyond its viscoelastic properties, exhibits antinociceptive and anti-inflammatory actions and has demonstrated both *in vitro* and *in vivo* disease-modifying effects beneficial in the preservation of the extracellular matrix. The current literature supports that HA is not only a medical device used for joint lubrication but a biologically active molecule that can improve the physiology of articular cartilage (Nicholls et al., 2017). Although viscosupplementation has been used for more than 3 decades and is recommended in several guidelines for the management of OA (Sellam et al., 2020; Olivier et al., 2019; Jordan et al., 2003; Rillo et al., 2016; Trojian et al., 2016), the use of viscosupplementation in OA remains a topic for debate regarding treatment efficacy and safety and the best dosing regimen. These discrepancies may originate from differences between the marketed HA products that differ widely from one to another (Webner et al., 2021; Altman et al., 2016). Optimizing clinical effectiveness of viscosupplementation is an exciting challenge for manufacturers, who develop new formulations adding various active molecules (antioxidant (Conrozier, 2018), corticosteroid (Hangody et al., 2018), non-steroidal anti-inflammatory drugs (Badawi et al., 2013), gold particles (Rasmussen et al., 2024), bisphosphonate (Scanu et al., 2023)) to HA.

In the present study we assess the analgesic and chondroprotective effect of a HA-tranexamic acid conjugate (HA/TXA) in an animal model of knee OA. TXA (Trans-4-amino-methyl-cyclohexane-carboxylic acid) is a strong and specific inhibitor of the plasminogen activator (PA) with inhibiting effects on the proteolytic enzyme through targeting of the fibrinolysis pathway (Vignon et al., 1991).

## Methods

### Material

The studied medical device (PANDORA^®^, LABRHA SAS, Lyon, France) is a sterile, non-pyrogenic solution of high molecular weight HA (3.5 MDa), of *streptococcus* equi biofermentative origin, (HTL-BIOTECHNOLOGY, Javene, France) and tranexamic acid (TXA) (SPECTRUM CHEMICAL, New Brunswick, New Jersey, USA) in a phosphate buffer of purified water, sodium chloride (NaCl), disodium phosphate (DSP, Na2HPO4) and monopotassium phosphate (MKP, KH2PO4) (COOPER, Melun, France). One milliliter of PANDORA^®^ contains 22 mg of HA and 15 mg of TXA. The PANDORA gel is indicated in the symptomatic treatment of knee OA and is the subject of a research program still in progress (clinicalTrials.gov Identifier NCT05414617 and NCT05978180).

The same HA, at the same concentration, diluted in the same phosphate buffer, was used as an active control.

### Ethical considerations

The animal experiments were carried out in compliance with European directive 2010/63/EU in accordance with French legislation (decree 87/848) at the GIP CYCERON/CURB (Centre Universitaire de Ressources Biologiques, approval #D14118001). The procedures were approved by the Ministry of Education and Research and the committee of regional ethics (CENOMEXA, France, APAFIS number #16185).

The manipulations were carried out following the ARRIVE guidelines (Animal research: report of *in vivo* experiments; https://www.nc3rs.org.uk). All animals were handled in accordance with recognized international ethical principles for the use of laboratory animals and every effort was made to refine the experiments, reduce the number of animals used and replace the use of animals when this was possible, and the experimenters were aware of their responsibilities.

### Animals

Fifteen C57bl/6J 8-week-old male mice from the Janvier Labs laboratory were used. The animals were housed in a room with controlled temperature and light (temperature 23°C ± 2°C, 12-h day/night cycle in reverse cycle) at CURB, University of Caen Normandy, France. The animals had access to food and water *ad libitum* and the cages were changed once a week. The experiments were performed between 8a.m. and 5p.m. in a room with dim illumination (6 lx). All the behavioral procedures were carried out within the HANDIFORM platform (UNICAEN, Caen, France).

### Induction of osteoarthritis

**FIGURE 1:**
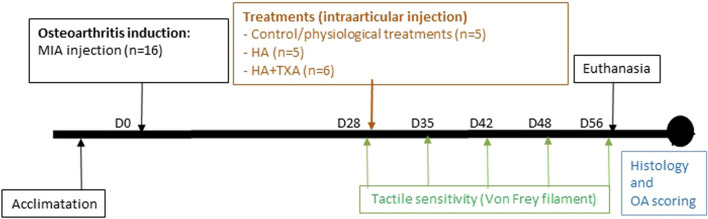
Timeline of the experimental procedure.

The experiments were performed according to the timeline presented in Figure 1.

Osteoarthritis was induced at day 0 by intra-articular injection of 0.75 mg of Monosodium IodoAcetate (MIA, Sigma-aldrich) dissolved in 10 μL of sterile physiological serum. Alcohol was used to disinfect the bench and a field was laid down. Animals were anesthetized using 5% isoflurane mediated by 70% N_2_O/30% O_2._ then maintained at 3% isoflurane (70% N_2_O/30% O_2_). Anesthesia time was on average 10 min, including recovery and induction time. The animals were placed in dorsal recumbency, and the right knee disinfected with 2% alcoholic chlorhexidine (Gilbert, Hérouville-Saint-Clair, France). The knee was bent to 45° and the joint was identified by observing the patellar tendon through the skin of the animal; the injection of 10 μL was then performed in 1 min into the synovial capsule using a 30 G needle (microlance 3 30 G 1/2″0.3 × 13 mm; BD, Rungis, France) and a 25 μL syringe (Hamilton, Giarmata, Romania). After removal of the needle, the joint was mobilized to diffuse the product. The injection site was cleaned with physiological serum in order to remove potential traces of MIA which could create skin inflammations. Finally, the anesthesia was lifted, and the animals were kept in the hand until fully awakening to limit the drop in body temperature. The health of the animals was monitored for 1 h after the injection without ever observing any sign of discomfort.

### Treatments

At day 28, according to the same protocol of MIA injection, the mice received a single intra-articular injection of one of the following treatments: Control-group (n = 5) received 10 µL of physiological serum, HA-group (n = 5) received 10 µL of hyaluronic acid, and HA/TXA-group (n = 6) received 10 µL of Pandora^®^.

### Functional pain outcomes

Tactile sensitivity was assessed using von Frey filaments (Ugo Basile, Salon-de-Provence, France).

The mice were placed in isolation cages placed on a raised grid. An exploration time of 30 min is given to the animals so that they are as calm as possible. Using nylon filaments, a controlled weight pressure is exerted between the pads of the animals’ hind legs. A response to the stimulus is counted if the animal withdraws its paw (snap, withdrawal or lick). A response is not counted if the animal moves accidentally at the time of stimulation. Both hind paws were tested 5 times in a random order and with a refractory period of 1 min between each trial. If three responses are counted over 5 trials, then the animal responds to the stimulus associated with the weight of the filament. Stimulations start at 1 g. If the animal does not respond to stimulation, the increase in stimulation applied will increase until a response is obtained (1.4 g, 2 g, 4 g). If the animal responds to the 1 g stimulation, the decrease in stimulation will be carried out until there is no response (0.6 g, 0.4 g, etc.). In the case of a reduction in stimulation, the filament retained is the last one to have provoked a series of three responses. To analyze these results, the ratio of responses to stimuli under the right paw is compared to responses under the left paw for each animal.

Before evaluation, the mice underwent 3 weeks of habituation to handling with one visit every 2 days, excluding cage changing days, in order to familiarize themselves with the handler and the handling and thus reduce their stress.

### Histological examination

At day 56, the mice were euthanized by cervical dislocation after deep anesthesia (5% isoflurane, 70% N2O/30% O2). The knee joints were dissected, fixed for 3 days in 10% neutral buffered formalin (NBF), and decalcified in Osteosoft (Sigma Aldrich) for 72 h week at room temperature and then left, cryoprotected by a 15% sucrose bath, for 48 h at 4°C. Thereafter, the knees were embedded in OCT and sections with 10 µm thickness were prepared using a cryostat (CM3050 S, Leica). Sections were stained with Safranin O and counterstained with Fast Green.

Cartilage degradation was assessed using the OARSI score (Pritzker et al., 2006). This score is separated into 6 grades, including changes in the subchondral bone for the last two. The different grades of the OARSI score according to Pritzker make it possible to precisely quantify cartilage degradation, with a score ranging from 0 to 6.5.

### Statistical analysis

Statistical analysis and graphs were performed on GraphPad Prism 8.0.2 software. The normality of all data was checked by the Shapiro-Wilk test and verified visually by a QQ plot. For non-parametric unpaired data, a Kruskal–Wallis test was performed as well as a *post hoc* test for two-by-two comparison of groups using the one-sided Mann-Whitney test. For unpaired parametric data, only two-group comparisons were performed and the t-test for unpaired data was used. The significance threshold was set at *p*-value <0.05.

## Results

### HA/TXA reduced OA pain

**FIGURE 2:**
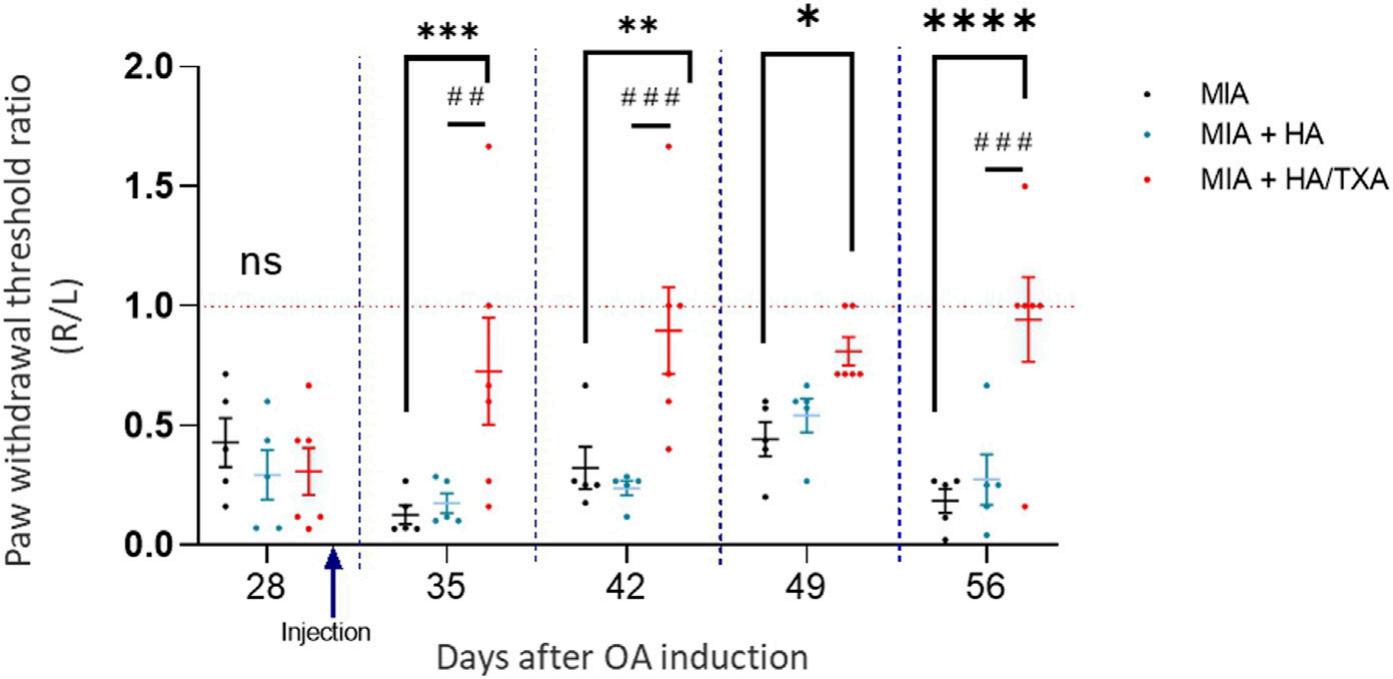
A single intraarticular injection of HA + TXA improved pain during osteoarthritis. Osteoarthritis (OA) was induced by intraarticular injection of MIA in the right knee of mice. Four weeks later, OA knees of mice were injected with physiological serum (n = 5), HA (n = 5) or HA + TXA (n = 5). Tactile sensitivity was evaluated in both paws at days 28 (before treatment injection), and then once a week for 4 weeks. Values are presented as ratio of withdrawal threshold between right and left paw (R/L). Data are expressed as means ± SEM. *p-value <0.05. **p-value <0.01.

On day 28 after OA induction, just before treatment administration, we observed no differences between the three groups in paw withdrawal threshold. Throughout the experiment and on day 56, we observed that animals given saline had a lower paw withdrawal threshold for the OA paw (right) than for the non-OA paw (left). Mice injected with HA presented the same profile, suggesting the absence of a beneficial effect of HA on OA pain. Conversely, HA/TXA administration increased the paw withdrawal threshold, indicating a reduction in tactile sensitivity of the arthritic paw and therefore a reduction in joint pain induced by OA. The significant difference in the paw withdrawal threshold after HA/TXA administration shows that the beneficial effect lasts at least 4 weeks after HA/TXA injection (Figure 2).

### Both HA and HA/TXA reduced OA progression

**FIGURE 3:**
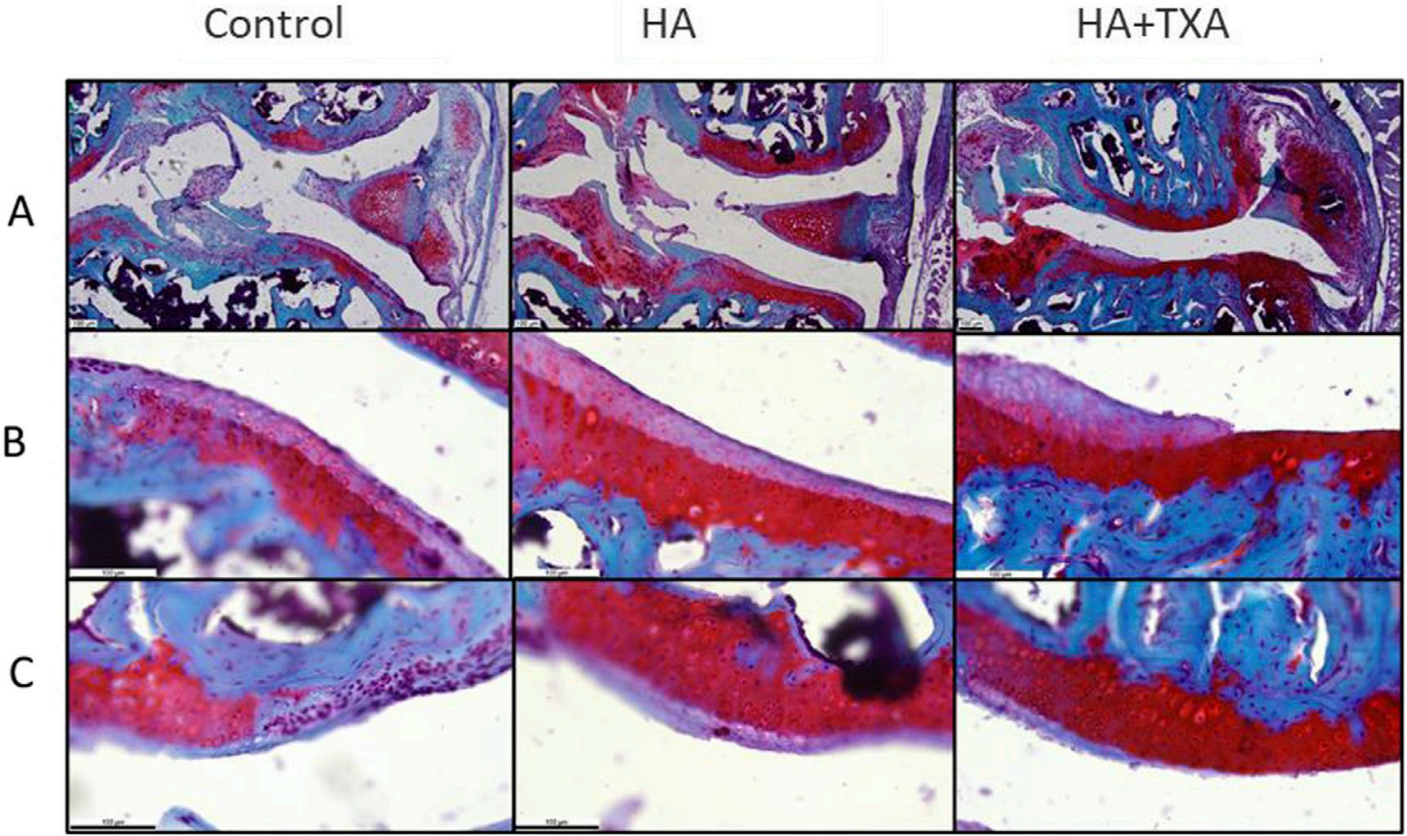
A single intraarticular injection of HA + TXA reduced OA progression in mice. Osteoarthritis (OA) was induced by intraarticular injection of MIA in the right knee of mice. Four weeks later, OA knees of mice were injected with physiological serum (n = 5), HA (n = 5) or HA + TXA (n = 6). Mice were euthanized 8 weeks after OA induction, and knee sections were stained with safranin O/fast green. **(A,B, C)** Representative images of knees. **(A)** Internal joint of the right knee of the mouse (where the lesions are located) **(B)** Cartilage of the internal tibial plateau, **(C)** Cartilage of the internal femoral condyle.

**FIGURE 4:**
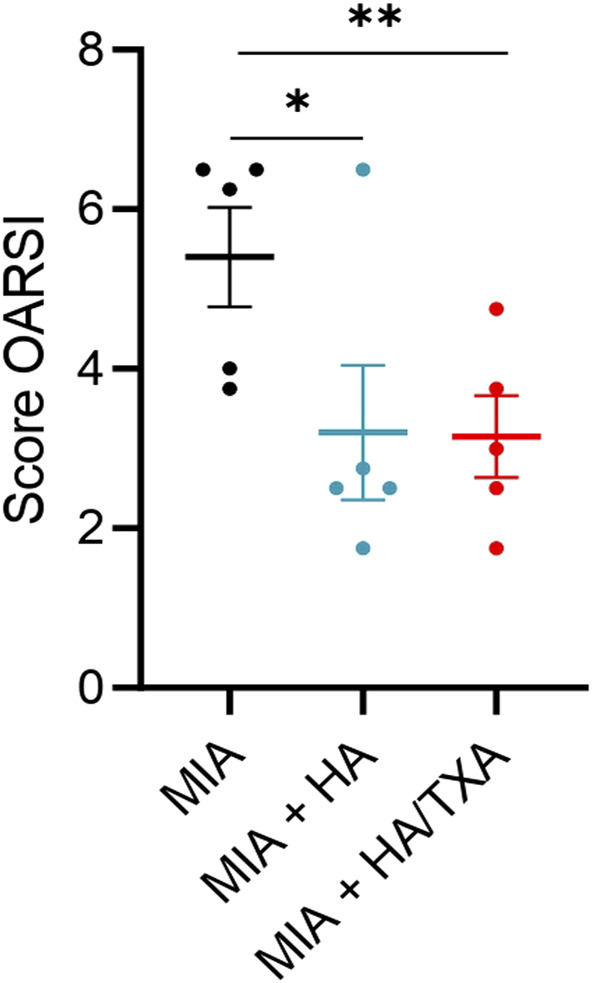
OARSI score at Day 56 after intra-articular injection of salie, HA or HA/TXA. Data are expressed as means ± SEM. *p-value <0.05. **p-value <0.01.

In the control group, we observed that MIA induced an OA of high grade (OARSI score = 5.5 ± 0.62). Interestingly, administration of HA and HA + TXA was able to reduce the OARSI score (score = 3.2 ± 0.84 and 3.15 ± 0.51, respectively), suggesting that both treatments are able to improve OA and reduce its progression (Figure3). Individual results are given in Figure 4.

## Discussion

This animal study suggests that the addition of TXA to a high molecular weight HA makes it possible to obtain an analgesic effect greater than that of HA alone, without reducing its chondroprotective effect.

Tranexamic acid is a lysine analog that binds with high affinity to the lysine binding sites (LBS) of plasminogen. Hence TXA prevents plasminogen activator (PA) activation and therefore plasmin production. Fibrinolysis is one of the major pathological changes occurring in arthritic joints, in the pathophysiology of which the PA system plays a key role. The balance between PA and plasminogen activator inhibitor (PAI) determines the direction in which plasminogen is activated into plasmin, which in turn further promotes fibrinolysis by activating MMPs (Masuko et al., 2009). Several other anti-osteoarthritic drugs interact with the PA system. The Receptor for u-PA, which is expressed in the synovium in both OA and rheumatoid arthritis, has been shown to be attenuated by treatment with HA (Masuko et al., 2009). The anti-inflammatory and analgesic effect of hydrocortisone is due, a least in part, to the upregulation of PAI-1 expression along with downregulation of tissue PA activity as demonstrated in both normal and inflammatory conditions (Nonaka et al., 2000). Tranexamic acid, from sub micromolar concentrations, attenuates the activation of plasminogen and considerably reduces the production of plasmin (Wu et al., 2019). It is mainly for its anti-fibrinolytic effect that TXA is used as an antihemorrhagic agent in cases of trauma, as well as in major surgical procedures, such as cardiac, orthopedic and hepatic surgeries (Pai et al., 2023). *In vitro* studies also suggested that plasmin may promote OA development through multiple mechanisms, including the degradation of proteoglycans and lubricin, and the stimulation of the synthesis of inflammatory and degradative mediators (Wang et al., 2024). The beneficial effect on pain of the TXA/HA combination is likely due to the anti-inflammatory properties of TXA. In patients treated with knee arthroscopic arthrolysis, the pain scores and the serum levels of proinflammatory mediators IL-6 and C Reactive-protein were shown to be significantly lower 1 week after surgery in patients who received topical administration of 500 mg of TXA at the end of the surgical procedure than that of those who did not (Li et al., 2023). Likewise, the TXA group underwent better post-operative range of motion and Lysholm knee scores (Nick and Deiary F., 2013).

In our experiment both HA and HA/TXA demonstrated a chondroprotective effect, as shown by the lower OARSI score compared to that of saline injection. The structure-modifying effect of HA has been widely evidenced (Henrotin et al., 2020). Exogenous HA increases the synthesis of extracellular matrix molecules, stimulates synovial fibroblasts to produce new HA, the amount of newly synthesized HA being dependent on both the concentration and molecular weight of the exogenous HA (Nicholls et al., 2017). Exogenous high molecular weight HAs have demonstrated a higher stimulating effect on HA synthesis than low molecular ones (Smith and Ghosh, 1987). HAs used in the present study were both of HMW with an average MW of 2 MDa. We were unable to demonstrate a greater chondroprotective effect thanks to the addition of TXA. However, several studies argue for an anti-osteoarthritic effect of TXA. In an animal model of OA, Wang et al. showed that the pharmacological inhibition of plasmin attenuated OA progression while injection of plasmin exacerbated OA (Wang et al., 2024). Many years ago, Vignon et al. assessed the effects of TXA in a rabbit model of OA induced by section of the knee anterior cruciate ligament. Prophylactic treatment administered intramuscularly thrice weekly for 3 and 6 months significantly inhibited stromelysin and collagenase activity and reduced cartilage destructive lesions (Vignon et al., 1991). Butler et al. showed, in rabbits subjected to partial lateral meniscectomy and section of the sesamoid and collateral fibular ligaments, that triamcinolone hexacetonide as well as TXA exhibited significant anti-osteoarthritic activity (Butler et al., 1983). Likewise, it has been demonstrated that TXA reduced urinary collagen cross-link excretion in both adjuvant-induced arthritis and rheumatoid arthritis (Ronday et al., 1998).

To demonstrate the benefit of TXA/HA treatment, we used monoiodoacetate to induce OA in the knees of mice. This chemical model is frequently used to exploring OA-related pain (Aüllo-Rasser et al., 2020; Upadhyay et al., 2023), which is the main reason OA patients seek medical help. Indeed, intraarticular injection of MIA induces rapid pain-like responses in the ipsilateral limb as well as morphological changes of the articular cartilage and bone disruption which are reflective of some aspects of patient pathology (Pitcher et al., 2016). Nonetheless, it does not recapitulate the natural onset of the human disease and does not reproduce entirely all symptoms of the disease (de Sousa Valente, 2019). In particular, the initiating events are not typical of OA. In addition, there are obvious limitations due to use of mice, particularly those related to differences in anatomy, gait, and cartilage characteristics compared to human joints. Thus, as all animal models, the murine MIA model only mimics parts or stages of the disease, but does not completely reproduce human OA complexity (de Sousa Valente, 2019). Nevertheless, it has been shown to have a pre-clinical value and has the advantage of generating robust and reproducible pain phenotypes associated with the alteration of joint tissues (Micheli et al., 2019; Bove et al., 2003).

Monoiodoacetate is a cysteine peptidase inhibitor that targets the glyceraldehyde-3-phosphate dehydrogenase (GAPDH), a key enzyme in the glycolysis pathway. The disruption of glycolysis in joint cells results in chondrocyte death, neovascularization, subchondral bone necrosis and collapse, as well as inflammation (de Sousa Valente, 2019). The death of chondrocytes makes it impossible to maintain the extracellular matrix, which deteriorates over time, leading to joint degeneration similar to that seen in osteoarthritis (Aüllo-Rasser et al., 2020).

Despite its artificial onset, MIA model offers several useful advantages compared to other murine models. For instance, this inflammatory chemical model is less invasive than surgical models (Alves-Simões, 2022) and consequently easier to induce and more reproducible, making it ideal for short-term trials. Furthermore, the MIA model offers a unique opportunity to study OA-related pain, whereas surgical methods, such as destabilization of medial meniscus or menisectomy are more useful for studying the onset and progression of posttraumatic osteoarthritis as well as the structural and biological processes throughout the progression of the pathology (Alves-Simões, 2022).

Because animals are nonverbal, assessment of pain is challenging. However, several methods have been proposed for monitoring pain in mice, including examining the animal directly and making a subjective assessment of animal comfort based upon hair coat, posture, general activity level, and degree of alertness (Turner et al., 2019). In the context of an experimental OA model, the manual Von Frey test, that we used in this study, is the most common method to quantify “pain-like” behaviors in mice (Drevet et al., 2022). This classical static test permits to identify mechanical/tactile allodynia. It can be used in the clinic to quantify tactile allodynia, and it can also be a useful diagnostic tool in preclinical animal experiments. In OA mice, the innocuous mechanical pressure is applied using nylon filaments on the hind paw as a measure of secondary mechanical hyperalgesia (Alves-Simões, 2022). Other tests exist; we can cite the static or dynamic weight-bearing assay to evaluate weight distribution asymmetry induced by pain; the rotarod to measure ambulatory pain, or the hot/cold plate test to measure thermal hyperalgesia (Alves-Simões, 2022) but they are more difficult to perform or less reproducible. Interestingly, the MIA model associated to pain assays has been pharmacologically validated by commonly used therapeutic agents such as paracetamol, naproxen, or diclofenac, which alleviated pain following MIA injection. In addition, the MIA model has been used in several preclinical OA studies which are currently continuing with phase IIa clinical trials (Alves-Simões, 2022), making it the most adequate model for this preclinical study aiming to investigate the chondroprotective and antinociceptive effects of HA-TXA.

## Data Availability

The raw data supporting the conclusions of this article will be made available by the authors, without undue reservation.
